# Simulated ward round training in the medical curriculum Munich

**DOI:** 10.3205/zma001471

**Published:** 2021-04-15

**Authors:** Christian Lottspeich, Leah T. Braun, Martin R. Fischer, Ralf Schmidmaier

**Affiliations:** 1 Ludwig-Maximilians-Universität München, LMU Klinikum, Medizinische Klinik und Poliklinik IV, Munich, Germany; 2 Ludwig-Maximilians-Universität München, LMU Klinikum, Institut für Didaktik und Ausbildungsforschung in der Medizin, Munich, Germany

**Keywords:** curriculum development, internal medicine, ward rounds, simulation course, teaching methods, medical education

## Abstract

Conducting a ward round in a structured and goal-oriented manner is one of the central competencies of a physician's work. Despite its relevance, ward round competence was only addressed in an unstructured way in the Medical Curriculum Munich (MeCuM) prior to 2011. Therefore, the project’s aim was to implement an evidence-based course on medical ward round competence. This project report provides a guideline for developing such a training course. Project planning and development was guided by the steps of the “Kern cycle”, beginning with needs assessment, learning objectives definition, and selection of appropriate teaching methods, and ending with implementation and evaluation.

## Zusammenfassung

Eine Visite strukturiert und zielführend durchzuführen gehört zu den zentralen Kompetenzen der ärztlichen Arbeit auf einer Station. Trotz der Relevanz wurde Visitenkompetenz im Medizinischen Curriculum München (MeCuM) bis 2011 nur unstrukturiert adressiert. Ziel dieses Projektes war es daher, einen evidenzbasierten Kurs zur Visitenkompetenz zu implementieren. Der vorliegende Projektbericht stellt einen Leitfaden zur Entwicklung eines solchen Kurses bereit. Bei der Projektplanung und -entwicklung wurde sich am Modell und Schritten des Kernzyklus orientiert, angefangen von der Bedarfsanalyse, über Lernzieledefinition und Auswahl angemessener Lehrmethoden bis hin zur Implementierung und Evaluation.

## 1. Introduction

Medical ward rounds are a central and complex part of the medical routine of any specialization and usually have to be performed independently from the first day of clinical activity. Conducting a ward round is an entrustable professional activity (EPA) [1], which is learned step by step in training and continued education. As part of a comprehensive reform of the Munich Medical Curriculum (MeCuM), a basic interdisciplinary clinical year with continuous interdisciplinary teaching, learning and examination was created [2]. In the course of this development, the need to explicitly teach ward round competencies was recognized. Therefore, the responsible curriculum planners used the "Kern cycle" [3] to develop a simulated ward round training course (“Simulierte Lehrvisite”; SiLVi) to meet this need. The purpose of this project report is to illustrate the process of curriculum development from needs analysis, learning objectives definition, and selecting of appropriate teaching methods to implementation and evaluation of the course. This will offer other curriculum planners a tool to systematically teach ward round skills at their own site according to their needs. We hope to be able to contribute to the improvement of ward round competencies beyond our own faculty.

## 2. Project description

### 2.1. Step 1: Problem identification and general needs analysis

Already on the first day of a work, it is expected that physicians carry out a ward round competently [4]. However, it is known from a large number of studies that young physicians sometimes have great problems in conducting ward rounds in a structured and goal-oriented manner [4], [5]. Inadequate ward round competence primarily affects the patients. Errors that can result from poorly managed ward rounds ultimately jeopardize patient safety [6], [7]. In addition, patient satisfaction also suffers from suboptimal ward rounds. Physicians themselves are also affected, who feel insecure and not up to their task [8]. Delays or ambiguities caused by an inexpertly conducted ward round can lead to conflicts with other professions, especially in nursing. It can be assumed that this has a negative impact on the quality of care, the length of stay of patients and the profitability of the hospital.

The reason for a lack of ward round competence is, on the one hand, the complexity of the ward round since various goals are pursued here and numerous domains of competences are required [9], [10]. On the other hand, it is known that the teaching potential of the ward round is rarely exploited, students do not participate in ward rounds and often do not receive supervision [11], [12], [13]. 

In Germany, young residents are likely insufficiently prepared for ward rounds during their studies. Although ward rounds are partly used to teach practical and theoretical content, they are rarely used to reflect on the ward rounds themselves. 

In the National Competence-Based Learning Objectives Catalogue for Undergraduate Medical Education (NKLM) [http://www.nklm.de] in Germany, ward rounds are listed as a competence and at the end of the final clinical year a competence level 3a (“perform and demonstrate under supervision”) is demanded. The teaching method for achieving this competence is left to the individual medical faculties, as is the case for all other content of the NKLM. 

In MeCuM, single domains of ward round competence were taught (especially in the field of communication) and students participated in ward rounds in an unstructured manner, but no explicit curricular course existed to date to systematically promote this competency.

#### 2.2. Step 2: Targeted needs analysis

A curriculum project to promote ward round competence can have different target groups: in addition to medical staff – chief physicians, senior physicians, residents, medical teachers, medical students at various levels of training – as well as nursing staff, psychologists, physiotherapists, and occupational therapists. A top-down strategy (i.e., first addressing the senior physician level (teach-the-trainer), then residents, then medical students) appears effective and sustainable, but is fraught with enormous implementation hurdles. Physicians with professional experience have generally learned ward round competence in an unstructured way and by adopting observed behaviors during ward rounds (imitation). Still they have reached a level of expertise with which they can adequately cover many aspects of ward round competence – at least in self-assessment. The lack of time for in-house training and structured ward rounds teaching as part of continuing education is a major obstacle. Therefore, the undergraduate medical student was defined as the targeted learner. Curricular mapping was performed to determine the specific needs of the students. It was intended to make clear to the curriculum planners – and also to the teachers and learners – how domains of competencies develop into a general ward round competency and how the competency can be further developed during the course of the curriculum. The prerequisite for the curricular mapping was the definition of the domains of competence for the professional activity “conducting a ward round”. A systematic literature review revealed that although various sub-competencies had already been explored in the literature, there was no coherent model of ward round competency [14]. A qualitative scientific study of ward round competency in internal medicine was therefore conducted by our group with expert interviews from different medical specialties, levels of care, expertise levels, and professions [14], defining nine competency domains with 18 individual competencies (see table 1 (Tab. 1)). A second interview study investigated two further ward round settings that were as different as possible - namely surgery and psychiatry - and was able to show that the defined ward round competencies differed only in subject-dependent weighting, but not fundamentally [15]. The competencies appear to be largely independent of medical disciplines. Table 1 (Tab. 1) shows the curricular mapping in MeCuM for the defined domains of ward round competences.

To summarize the curricular mapping presented above and domains of ward round competencies extracted by means of the interview study, the following specific needs could be identified:

- the acquisition of professional knowledge [16], [17] to ward round competence,
- a tool for feedback and self-reflection as a prerequisite for self-directed, lifelong learning in the area of ward rounds skills and
- a structured hands-on training of the professional activity “conducting a ward round” with a focus on error management, self-management, leadership and organizational skills.

#### 2.3. Step 3: Learning objectives 

To improve ward round competency on the first day of practice as a physician, a course should be implemented in MeCuM with the overall learning objective:

*MeCuM graduates will be able to conduct and demonstrate a ward round under supervision.*

To meet the above needs, a training course for the clinical semesters was planned. This course should bring together previously acquired knowledge, skills, and learned behaviors of the domains of competence to perform a specific activity “conducting a ward round”. The course furthermore should supplement these competencies with specific professional knowledge, and create the ability and willingness for continuous development in the remainder of the medical studies and post-graduate training. The learning objectives are shown in table 2 (Tab. 2).

#### 2.4 Step 4: Teaching methods 

**Figure 1:**
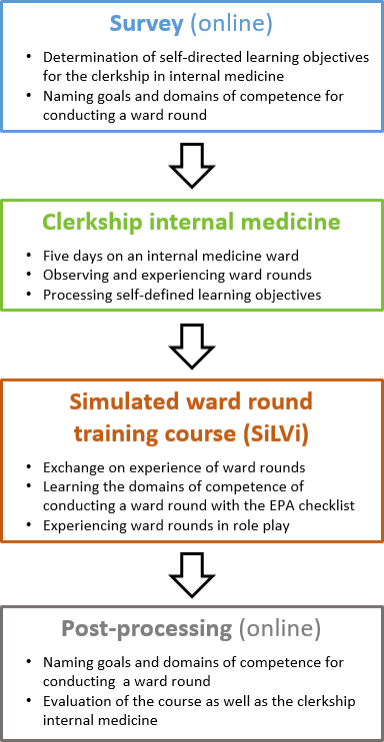
Structure of the clerkship in internal medicine (“Blockpraktikum Innere Medizin”)

The planned training course should take place in the basic interdisciplinary clinical year of MeCuM (6^th^ and 7^th^ semester; approx. 550 students per academic year). The new ward round course should be established in the compulsory curriculum and linked to the existing internal medicine clerkship (*“Blockpraktikum Innere Medizin”*). The structure of the clerkship in internal medicine with integration of the ward round training course is shown in figure 1 (Fig. 1).

Students were asked to observe and reflect on real ward rounds during their one-week clerkship (“real-life experience”) and then to implement the unstructured acquired knowledge in the SiLVi course in a structured manner through a simulated experience. In addition to the focus on ward rounds, students pursue self-defined learning objectives on the days they are present on the ward in the internal medicine clerkship in order to meet their individual learning needs (self-directed learning [18]). Students are free to choose the ward and thus their specialized focus for their clerkship through an online booking system.

Fifteen students per group take part in the SiLVi course after the clerkship in internal medicine. The ward round courses are led by a team of ten trained medical teachers. The course duration is 120 min. In the course, the EPA checklist is used, which was developed on the basis of the interview studies [15], [19] in a structured method by a team of experts and validated on the basis of 14 filmed real ward rounds [20]. The checklist is freely available as a supplement to the aforementioned work.

The timing and content of the SiLVi course, together with the assigned learning objectives and teaching methods, are shown in table 3 (Tab. 3). A teaching format was chosen for the ward round course in which learners could act realistically and actively in an environment typical of the workplace. The implementation of the course as a bedside teaching course was initially discussed by the curriculum planners. However it was decided to use a simulated ward round course and not a training course in hospital, due to the following reasons:

1. the medical complexity of the patient cases should be standardized as well as adapted to the prior knowledge of the students,
2. in the clerkship in internal medicine, the students already participate in real ward rounds and can bring this experience into the simulation,
3. the course in the form of a simulation offers the students a protected setting – the student takes on a role that they can discard after the scenario. Mistakes should be allowed and serve as a learning opportunity,
4. after observing the ward round in the clerkship and experiencing a ward round in the simulated role play, the students should then in the practical year conduct real ward rounds under supervision in the spirit of the learning spiral.

The course was implemented in the teaching clinic (“Lehrklinik des Zentrums für Unterricht und Studium”; ZeUS). Here, realistic patient rooms with video transmission to the neighboring seminar room and with Venetian mirror are available. 

In the training course, students have the opportunity to experience different perspectives of the ward round in role plays and to apply what they have learned. At the beginning of each scenario, all students are presented with the framework of the patient case (briefing). In addition, separate role scripts are handed out to the participants of the ward round as well as to the patients. In order to make the role play as realistic as possible, the participants wear appropriate professional clothing (gown, jacket) and patient charts (incl. medication list, vital signs) are available to the team. The observing students follow the ward rounds and evaluate them using the EPA checklist. 

The ward round scenarios were developed by medical experts and tested with medical personnel. In terms of content, the scenarios are based on ward round cases with a low level of medical complexity mainly because of the partly heterogeneous prior knowledge of the students. Frequently, ward round teams are focused on medical aspects of a patient case – in the course and accordingly to the developed ward round scenarios – however, the students were to focus primarily on further ward round competencies and less on medical expertise. The goal was to minimize problems caused by students’ lack of medical knowledge in the ward round simulation. The duration is approximately 5-10 minutes per ward round scenario. According to our research, this is a realistic time frame (4-12 minutes/patient case) [15], whereas the ward round duration per patient depends on the complexity of the individual patient case (simple - about 8 minutes; complex - about 18 minutes) [20]. For illustration purposes, a ward round scenario with the corresponding role descriptions is available as an online attachment (see attachment 1 ).

Potential conflicts are inserted in each of the ward round scenarios, so the ward round team has to respond accordingly. Examples of the conflicts in the scenarios are:

- pending test results (organizational issue)
- ward round participation of relatives (social competence)
- newly emerged symptoms of the patient (medical problem)

Following each ward round scenario, the actors reflect on their performance and the observers provide feedback with the help of the EPA checklist. Moderation and debriefing are done by the instructor.

#### 2.5. Step 5: Implementation

In 2011, a curriculum reform of the clinical years of study at LMU took place with the implementation of a basic clinical year (“module 23”). In the process, the disciplines of internal medicine (previously “module 2”) and surgery (previously “module 3”), which had previously been taught separately, were restructured into a total of eight organ-based modules and now taught on an interdisciplinary basis. Each of these organ-based modules (e.g. “module gastrointestinal system") comprises four weeks, which are concluded with a final exam.

As part of the reform, structure and content of the clerkship in internal medicine were also adapted and further developed. The conceptualization and implementation of the ward round training course as part of the clerkship was done by persons who were also part of the central organization team of “module 23” (RS, CL, among others). Thus, the curriculum reform of the clinical years of study section facilitated the implementation of the SiLVi course. After presentation of the state analysis as well as the need for a ward round training course, the project received broad support from the Dean of Studies, “module 23” spokespersons at the time as well as the students involved in the curriculum reform. Personnel costs were incurred primarily during the development phase of the project. No real costs were incurred by the project team due to the teaching commitments and medical didactic research activities of the curriculum planners. The costs for student assistants were covered by a teaching innovation funding instrument of the LMU Medical Faculty (Hildegard-Hampp-Humanitas Trust). There were no technical or construction costs, as simulation rooms for new teaching formats were available with the establishment of the teaching clinic (2011). The teaching clinic enabled the ward round course in its current form with simulation in a realistic patient room. 

The first concept for the training course was piloted with students in their practical year and residents. Their feedback helped to refine the content of the course. The lecturers were intensively trained and actively involved in the course development. This also enabled content-related and organizational problems to be identified prior to implementation. After optimizing the course schedule and adding further ward round scenarios, the first SiLVi courses were held in the summer term of 2012. In following courses, the concept was adapted by means of regular evaluations (by students and lecturers). 

When designing the training course, the possibility of using actors who play standardized patients was discussed. This could have made the ward round situations more controllable and the process more predictable. However, role-playing offered students the change to take on the patient-role – an experience that was likely new to most students. In the opinion of the course developers, the advantage of role-playing outweighed that of standardized patients, which would also have involved additional financial and personnel costs. The active experience of the patient role was also supported by the students and evaluated as very positive in the course evaluations. 

Regarding the course content, there was concern prior to implementation that students would not volunteer to participate in the role plays. Therefore, it was mandated that each student must actively participate in a scenario at least once. This was ultimately well accepted by the students. For individual cases, the option of a written reflection paper as a substitute performance was kept open. In the briefing it is emphasized that the course takes place in a protected setting and that mistakes can be made and that a duty of confidentiality applies. Students are taught that this is precisely how a lasting learning effect can be achieved. 

Physicians who were judged by the course developers to have sufficient experience in ward rounds were requested individually to be instructors of the course. Medical teachers were either specialist for internal medicine or were internal medicine residents. The residents had to have sufficient ward round experience (at least one year of professional experience with participation in the hospital's ward round system). In addition, they should also have the educational competence to adequately lead the course and moderate the discussion/feedback rounds. Initially, each course was conducted by two teachers: one physician took over the content-related course leadership, and one psychologist or pedagogue managed moderation and debriefing. Students reported back that it was especially important for them to be accompanied by a physician trainer during the facilitation and debriefing process. The physician could guide them in transferring this somewhat ideal world of simulation to the real world of medical ward rounds. Subsequently, the course was only led by physicians.

In order to ensure a high quality of teaching, the instructors were briefed in advance by the course developers in form of structured individual training sessions. In addition to teaching the theoretical basics of ward round competence, instruction on the course procedure (including technical aspects of video/sound transmission) was provided in the teaching clinic. In addition, medical teachers were trained in the basics of feedback and debriefing. New instructors participated as observers in at least one course. This was followed by a meeting to discuss possible challenges in the course and possible ways of dealing with these situations. At least the first course unit was accompanied by an experienced supervisor. In addition to the professional and content-related qualifications of the instructors, personal contact was also important to us in order to be able to assess the instructors' self-motivation and interest in the topic of ward rounds. Despite the complexity of the course, a homogeneous teaching quality was to be maintained. It was therefore decided to recruit teachers exclusively from our own internal medicine clinic and from the Department of Medical Education. This ultimately proved more challenging in recruiting new instructors, but still ensures short lines of communication and honest feedback loops.

## 3. Results

The SiLVi course has been offered since the summer term of 2012 and has been completed by 3466 students to date (as of 04/2020). The course was offered as a voluntary course after a trial period of two semesters. Thereafter, it was implemented as a mandatory course as part of the clerkship in internal medicine and has continued to date. Now that the course is mandatory, almost 100% of students have participated in the course. On average, 235 students have participated in the course per semester since the winter term 2014/2015. 

Even though the overall learning objectives and general course structure have not changed over the years, the SiLVi course has been adapted based on the research findings of the working group [14], [15], [20] to meet modern curriculum design in the sense of evidence-based medical education. Among other things, the EPA checklist was included in the course. An originally included instructional video showing a ward round simulation was later removed from the course in favor of the role plays due to time constraints. 

The SiLVi course in its current form (as of summer term 2019, winter term 2019/2020) was evaluated by both instructors and students:

1. students evaluated the simulation in terms of authenticity and were able to rate the course at the end using a scale.
2. instructors evaluated the course in terms of its authenticity.

**Figure 2:**
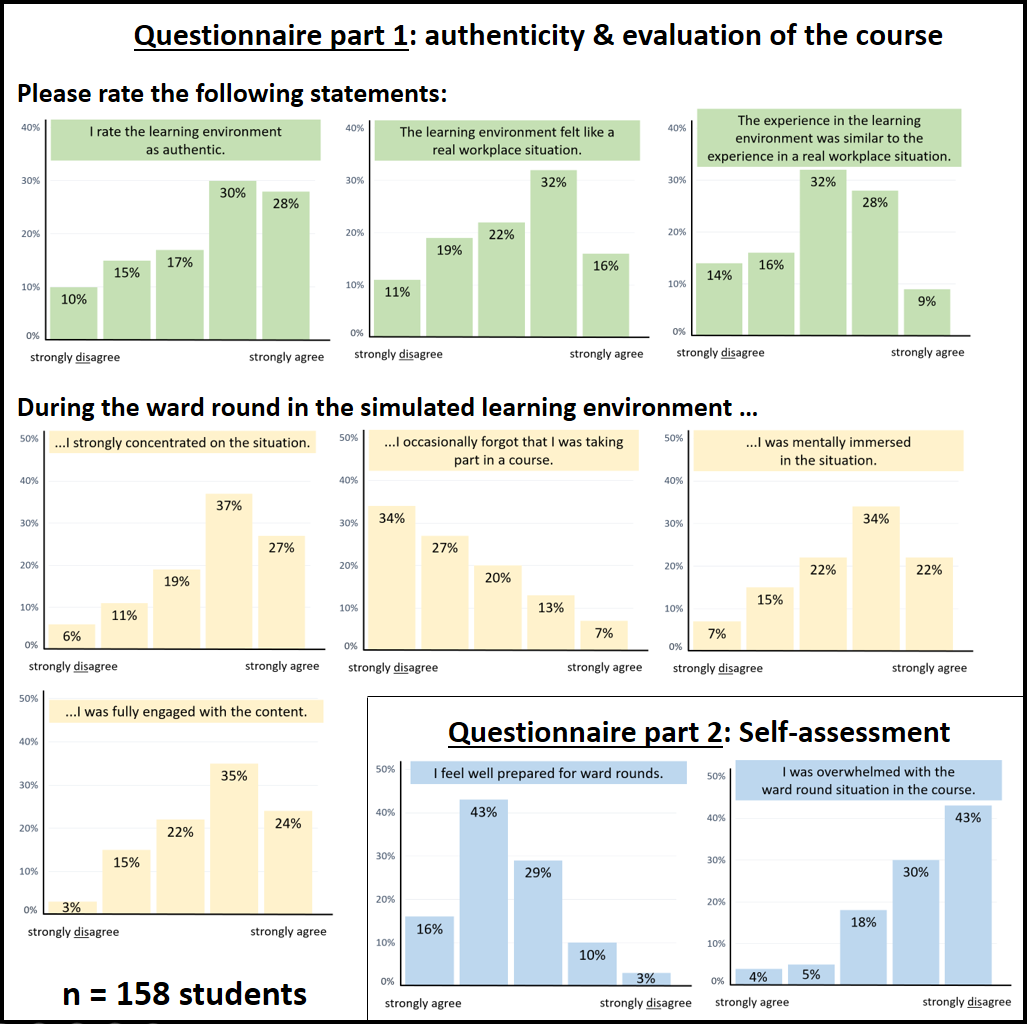
Authenticity and evaluation of the simulation course

**Figure 3:**
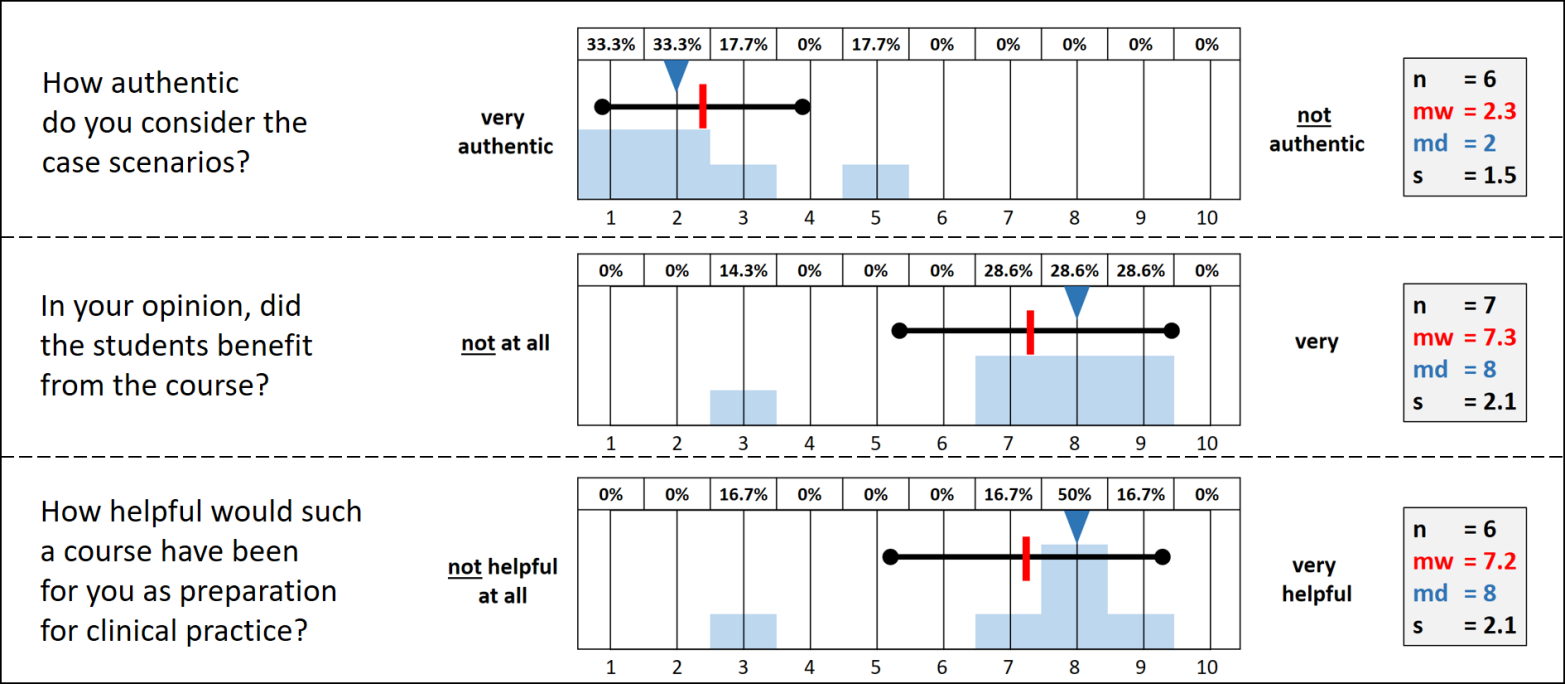
Evaluation of the simulation course by the instructors

The questions and their evaluation results are shown in figure 2 (Fig. 2) and figure 3 (Fig. 3). 

Overall, it was found that the students experienced the simulation as mostly authentic, which is an important evaluation parameter of a simulation environment. For the most part, they engaged with the simulation. Students did not feel overwhelmed by the course and felt well prepared for ward rounds. The course was rated an average of 2.2 on a scale of 1-5 (M=2.2±1.1). 

The instructors also felt that the course was authentic. In addition, they stated that the students benefited from the course and would have liked to have this course during their studies as preparation for later medical practice as well. 

## 4. Discussion

In the course of the project development and the further scientific work of the research group, it became apparent that ward rounds - although its relevance is undisputed - have so far been addressed little or not at all, not only in the medical curriculum but also in residency training. Our course is a partial component on the long-term path to creating a ward round curriculum that should ideally extend into residency training. In addition to a ward round curriculum for medical school, a structured implementation in the clinical internship year would be useful. Furthermore, we would advocate the inclusion of ward round competence as a training objective in residency training. Since ward round competence is an EPA, a workplace-based assessment would be valuable to complete the important steps from a simulated ward round (students), to a ward round conducted under supervision (young residents), to the independent and autonomous conduction of ward rounds (specialist). 

In view of the current evaluation of the project, a pre/post measurement was introduced as a new form of evaluation to further confirm the teaching concept. For this, students had to state the objectives of a ward round in an online survey both before and after the course. In the future, for long-term monitoring of learning success and for testing practical competence, external assessment should ideally take place, for example in the form of an OSCE (objective structured clinical examination).

The medium-term goal must remain to create corresponding projects for other training levels in the sense of a learning spiral. Finally, there is the additional possibility of promoting the teaching competence of clinical teachers through a train-the-trainer course. 

Further follow-up projects should focus on interdisciplinary collaboration (e.g., with nursing) and collaborative clinical reasoning projects (fewer wrong decisions with competently managed ward rounds).

## 5. Conclusion

In summary, after a corresponding needs analysis, the SiLVi course was successfully and sustainably implemented into the existing curriculum. The simulation was experienced by the students – as well as by the teachers – as an authentic situation and has already been completed by 3466 students (as of 04/2020). Since the ward round is a central part of daily work, a corresponding curriculum should be developed to optimally prepare students for their future work situation. Because of the many domains of competence, a longitudinal curriculum would be useful in which the individual competencies are first taught and then combined in a simulation course. This could be followed by transfer of practice with a teach-the-teacher course for clinical instructors and, in the long term, an EPA examination during residency training.

## Acknowledgements

Special thanks go to Dr. Stephanie Keil and Dr. Sophie Niedermaier-Patramani, who helped conceptualize the course and were crucial in its initial implementation. Furthermore, we would like to thank all participants of our research projects on ward round competence - the research results provided the opportunity to further develop the course in an evidence-based manner.

We thank all the instructors of the SiLVi course for their commitment and constant feedback. We also thank the students for their course participation and feedback on the course. 

Furthermore, we would like to thank for the financial support of the project initiation by the Hildegard-Hampp-Humanitas Trust.

## Competing interests

The authors declare that they have no competing interests. 

## Supplementary Material

Ward round scenario with the corresponding role descriptions

## Figures and Tables

**Table 1:**
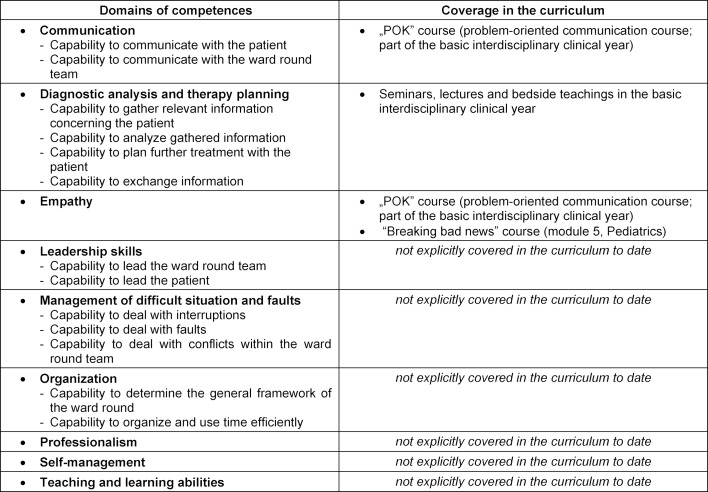
Domains of competence and curricular mapping of ward round competence in MeCuM [15]

**Table 2:**
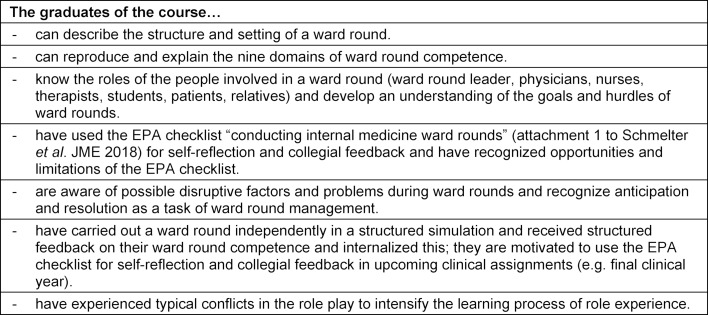
Learning objectives of the simulated ward round course (SiLVi)

**Table 3:**
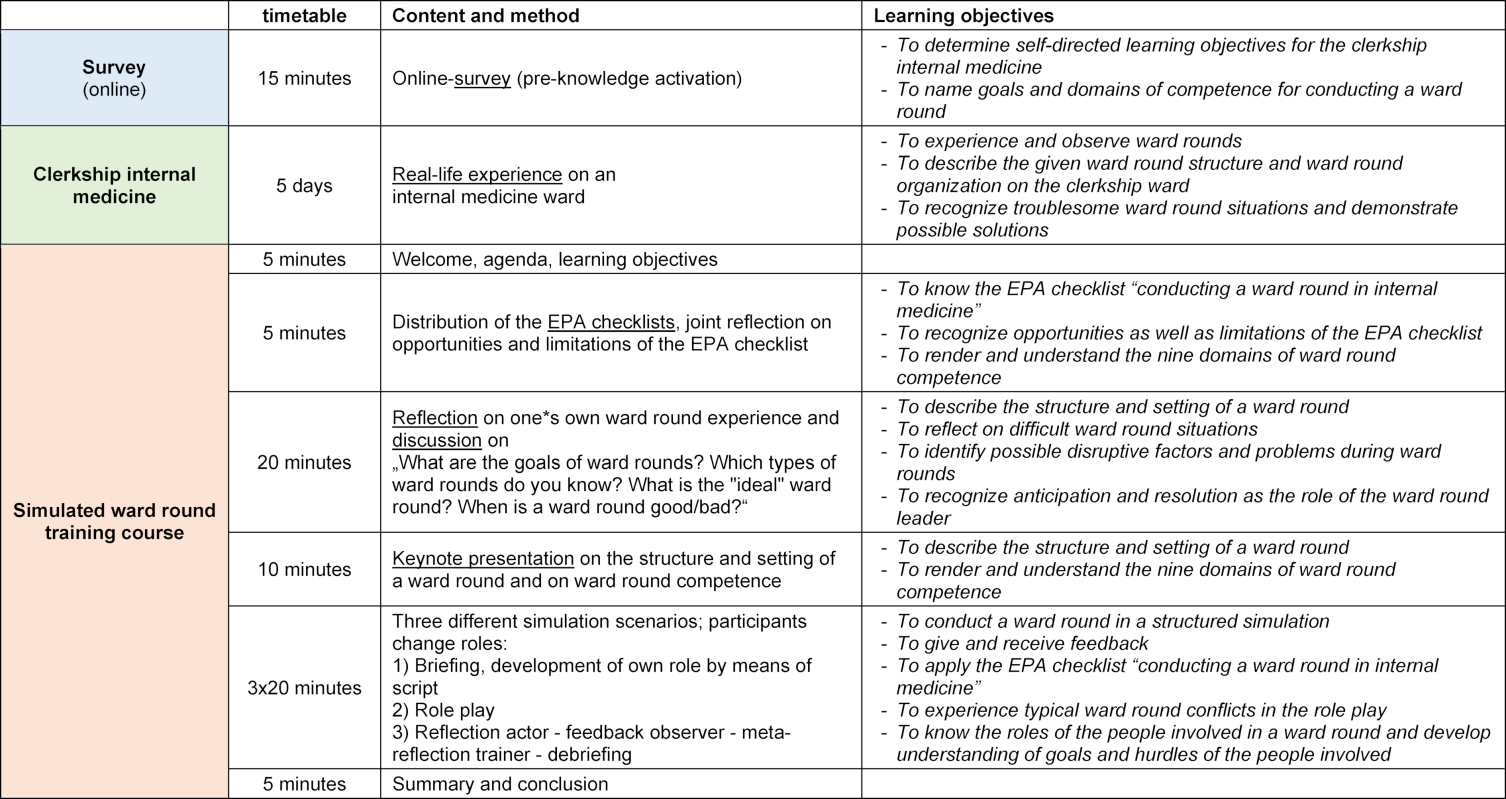
Program and learning objectives of the simulated ward round training course
