# Supplementary material for: Simulated ward round training in the medical curriculum Munich
Source: GMS J Med Educ. 2021 Apr 15;38(4):Doc75. doi: 10.3205/zma001471 (PMC8136345; doi:10.3205/zma001471)
Supplement: Ward round scenario with the corresponding role descriptions [file JME-38-4-75-s-001.pdf]

Case B

Mr./Mrs. White (75 years) was admitted to the hospital with a cough and progressive dyspnea. Respiratory symptoms had worsened and the patient was admitted to the hospital as an emergency. Tests ultimately revealed an exacerbation of a newly diagnosed obstructive pulmonary disease. The patient has been in hospital for three days, and clinical symptoms have already improved.

On admission, the chest X-ray revealed a pulmonary nodule in the right upper lobe. Therefore, further radiological diagnostics by computed tomography of the thorax was planned.

Attachment 1 to: Lottspeich C, Braun LT, Fischer MR, Schmidmaier R. *Simulated ward round training in the medical curriculum Munich.* GMS J Med Educ. 2021;38(4):Doc75. DOI: 10.3275/zma001471

Role description patient

You are Mr./Mrs. White and 75 years old. For several months, you have had this annoying irritating cough. About a week ago, you also noticed shortness of breath. The shortness of breath then worsened and you were admitted to hospital 3 days ago. Actually, you felt very healthy until now. Except for an appendicitis about 20 years ago, you have no previous illnesses. You have been smoking about two packs a day for 30 years.

You have been in the hospital for three days now. The doctors have prescribed two different sprays and several tablets for you to take regularly. In addition, you need to inhale several times a day. You already feel a significant improvement of your breathing problems. The doctors strictly advised you not to smoke, but today you secretly smoked your first cigarette - and you really enjoyed it.

Since your admission, various examinations have been performed, including a lung function and a chest x-ray. This x-ray showed a "nodule in the lung". This morning you went to the radiology department to have a computed tomography scan of the lungs. The examination was very quick - you would have imagined it to be more complicated. The doctors have not seen you yet today, so you will ask for the results of the examination during rounds. Also you had blood drawn this morning, so you'll be interested to know if the signs of inflammation have improved yet - they were "significantly elevated" on the day of admission. When will you be allowed to go home?

Today your wife/husband finally managed to come by. She really wanted to talk to the doctors - you don't really know why...

Attachment 1 to: Lottspeich C, Braun LT, Fischer MR, Schmidmaier R. *Simulated ward round training in the medical curriculum Munich.* GMS J Med Educ. 2021;38(4):Doc75. DOI: 10.3275/zma001471

Role description resident

Yesterday you had your day off after an exhausting night shift. Today you come back to work on your internal medicine ward. Your colleague has left you a note with the most important information about the patients, as he is on vacation since today.

Mr./Mrs. White had a CT scan and a lung function (for evaluation of the effect of therapy). The patient was admitted to the emergency room 3 days ago for an exacerbation of a chronic obstructive pulmonary disease. The hospitals internal computer system did not contain the results of the computed tomography prior to the ward round. Unfortunately, today you have not been able to visit Mr./Mrs. White yet. You hope that the student has already taken the blood sample and brought it to the lab.

Role description senior physician

You are a senior physician on the internal medicine ward and just arrived for ward rounds. You know Mr./Mrs. White for several days now. He has an exacerbated chronic obstructive lung disease and pulmonary nodule on the chest X-ray. This morning you looked at Mr./Mrs. White's X-ray again and are eagerly awaiting the results of the computer tomography you ordered yesterday. You hope that the CT scan will not confirm your suspicion that the lung nodule is malignant.

Attachment 1 to: Lottspeich C, Braun LT, Fischer MR, Schmidmaier R. *Simulated ward round training in the medical curriculum Munich.* GMS J Med Educ. 2021;38(4):Doc75. DOI: 10.3275/zma001471

Role description student

You are in the final year of your medical studies. You goal is to become a cardiac surgeon. Nevertheless, you are trying to get involved in the daily routine on the internal medicine ward. In the last few months, you have learned that the ward rounds are a good opportunity to obtain information. This morning you have already seen Mr./Mrs. White and took a blood sample. You think that the patient's general condition has already improved significantly. You wonder whether the patient now has a chronic obstructive pulmonary disease or asthma. How do you tell the difference?

Attachment 1 to: Lottspeich C, Braun LT, Fischer MR, Schmidmaier R. *Simulated ward round training in the medical curriculum Munich.* GMS J Med Educ. 2021;38(4):Doc75. DOI: 10.3275/zma001471

Role description nurse

From your point of view Mr./Mrs. White - a patient with exacerbation of a chronic obstructive pulmonary disease - has clinically already improved very much. You have been caring for him/her since admission. The pulmonary obstruction has already significantly improved due to medical therapy - and of course because of your good care.

Today you are a little bit upset, because you saw Mr./Mrs. White smoking outside the building. Your colleagues told you that Mr./Mrs. White might have lung cancer - in which case he should definitely stop smoking. You plan is to address this today on the ward round.

Tonight, Mr./Mrs. White slept very well. In the morning, the patient went to the radiology department for a CT scan. He just returned to the ward. To the best of your knowledge, the results are still pending.

Attachment 1 to: Lottspeich C, Braun LT, Fischer MR, Schmidmaier R. *Simulated ward round training in the medical curriculum Munich.* GMS J Med Educ. 2021;38(4):Doc75. DOI: 10.3275/zma001471

Role description husband / wife

You are the wife/husband of Mr./Mrs. White. For months, your husband/wife had this terrible cough. You had already told him/her a dozen times that he/she should go to the doctor - and above all, he should stop smoking. At night, the coughing was particularly bad. Therefore, you did not sleep very well the last weeks. Three days ago, the shortness of breath of your husband/wife became very bad. Therefore, you called the ambulance and he was taken to the hospital. You noticed a loud whistling sound that your husband/wife made when breathing out. You wanted to ask the doctors what this whistling sound was. Thank God, your husband's/wife's breathing problems are much better now.

You did not understand whether the current problem is a pneumonia or something else. Your husband/wife has not been able to explain it to you, so you are glad to finally meet the doctors today. Now you will be able to ask questions. You would be happy if your husband/wife could stay in the hospital for a few more days. You want him to get healthy again - maybe he/she could also go to a rehabilitation facility?

Attachment 1 to: Lottspeich C, Braun LT, Fischer MR, Schmidmaier R. *Simulated ward round training in the medical curriculum Munich.* GMS J Med Educ. 2021;38(4):Doc75. DOI: 10.3275/zma001471
